# Analysis of clinicopathologic characteristics and prognosis of gastric cancer in patients <40 years

**DOI:** 10.1097/MD.0000000000034635

**Published:** 2023-08-25

**Authors:** Lifeng Liu, Jina Lin, Jingrun Zhao, Peng Yan

**Affiliations:** a Department of Gastroenterology, Liaocheng Hospital, Shandong province, China; b Fuxing Hospital, Captital Medicial University.

**Keywords:** clinicopathological feature, gastric cancer, prognosis, survival, young patients

## Abstract

An increase trend and a bad prognosis toward gastric cancer (GC) in individuals <40 years have been observed over the past few decades. GC in Young adult needs further evaluation to identify clear risk factors for early screening and better prognosis. A total of 126 young adult patients with gastric cancer (individuals <40 years) (YG) were collected in Liaocheng hospital in China from Jan 2003 to Dec 2019. The overall median follow-up was 96.5 months (rang 1–192 months). Survival was determined by the Kaplan–Meier method and the difference in survival among different subgroups were assessed using the log-lank test. Correlations between risk factors and overall survival were assessed by univariate and multivariate Cox proportional hazards regression analysis. Advanced stage cancer at onset and undifferentiated histologic tumor type were the prominent clinicopathological features of YG. The 5-year overall survival of the YG was 31.7%. The 5-year survival of the YG differed from tumor staging and treatment methods. The 5-year survival was 100% in stage I group, 58.8% in stage II group, 22.6% in stage III group, and 8.3% in stage IV group respectively. The 5-year survival was 52.1% in the curative resection group versus 3.8% in the non-curative resection group. Multivariate analysis displayed that tumor staging (*P* = .002) and treatment method (*P* = .034) were 2 independent prognostic predictors for survival. GC in young adult patients have unique clinicopathological features. Upper gastrointestinal endoscopy should regularly perform for young adult especially those symptomatic patients. Early diagnosis and then proceed to a successful curative resection are vital for a better prognosis.

## 1. Introduction

Gastric cancer (GC) is an aggressive malignancy that carries a poor prognosis in the world.^[1–3]^ GC is one of the most commonly diagnosed cancers and a common cause of cancer-related deaths in China according to the 2018 Global Cancer Statistics.^[1,4]^ GC usually occurs in individuals aged 50 to 70 years, rarely occurs in individuals <40 years. Although a steady decline in GC has been observed, an increase trend toward GC in young adult (individuals <40 years, YG) has display over the past few decades.^[5–7]^ Some studies^[8–11]^ have evaluated the clinicopathological characteristics and outcome of YG. Generally, some characteristics such as female predominance, a higher frequency of diffuse tumor, positive family history, middle gastric location, diffuse histology type, shorter duration of symptoms and advanced stage seem to be typical.^[5,6,8–18]^ Studies indicated that YG had a similar or worse prognosis compared with middle-aged or older patients.^[5,7,9,11]^ YG are difficult to diagnose on the early stage because majority cases are asymptomatic or mild. YG need further evaluation to identify clear risk factors for early screening and better prognosis.

## 2. Methods

### 2.1. Study design

We carried out a single-center, retrospective, observational study in Liaocheng hospital in China. A total of 126 YG was collected from Jan 2003 to Dec 2019. All patients were histologically confirmed according to the International Classification of Diseases for Oncology^[19]^ and patients were excluded when a tumor other than primary GC (adenocarcinoma) was histologically identified. We classified the cases into 2 subgroups based on anatomic localization:^[20]^ cardia and non-cardia (gastric antrum, pylorus, lesser curvature and greater curvature of the stomach). The gross tumor appearance is categorized into 5 types: type 0 (protruding or superficial), type I (mass), type II (ulcerative), type III (infiltrative ulcerative) and type IV (diffuse infiltrative). Type 0 is subdivided according to the macroscopic classification of early GC. The histological classification of gastric tumors is categorized into papillary adenocarcinoma, tubular adenocarcinoma, poorly differentiated adenocarcinoma (pro), signet-ring cell carcinoma and mucinous adenocarcinoma. Signet ring cell carcinoma, pro and mucinous adenocarcinoma were defined as undifferentiated tumor. Papillary adenocarcinoma and tubular adenocarcinoma were defined as differentiated tumor. Tumor stages were classified as stage I, stage II, stage III and stage IV according to T-category (T), lymph nodes metastasis (N) and distant metastasis (M) stage. Tumors macroscopic types, histology and staging were classified according to the guidelines of the Japanese Classification of Gastric Carcinoma (Japanese Gastric Cancer Association).^[21]^ Patients were divided into curative resection group and non-curative resection group according to disease respectability based on TNM staging after evaluation of GC. Patient demographics data and tumor characteristics were collected using a uniform questionnaire. Patient demographics data included age, gender, family history of GC and clinical symptoms. Tumor characteristics included location, macroscopic types, histological type, tumor staging, treatment method. We used outpatient or telephone follow-up to understand the survival of patient situation. Patients were followed up every 6 months in the first 3 years and every year thereafter. The deadline of follow-up was June 2020.

### 2.2. Statistical analysis

Patients’ characteristics were presented with descriptive statistics such that continuous variables were delivered as range, whereas categorical variables were expressed as frequency (percentage). Survival was determined by the Kaplan–Meier method and the difference in survival among different subgroups were assessed using the log-lank test. Correlations between risk factors and overall survival were assessed by univariate and multivariate Cox proportional hazards regression analysis. Variables that were found to be statistically significant by the univariate analysis were further scrutinized in the multivariate analysis. A *P* value <.05 was considered statistically significant. All analyses were performed using the SPSS 17.0 (SPSS Inc., Chicago, IL) software.

## 3. Results

### 3.1. Patient characteristics

The clinical and histopathologic features of the YG were shown in Table 1. Among the 126 patients, there were 68 males (53.97%) and 58 females (46.03%). The age ranged from 20 to 40 years. Nine patients (7.14%) had a positive family history of GC. The clinical symptom included epigastric pain, dyspepsia, nausea/vomiting, anemia/bleeding, weight loss, heartburn/acid reflux and choking when eating. Epigastric pain (74.60%) and dyspepsia (58.73%) were most common symptom. Regarding to tumor location, most tumors located in non-cardiac position (91.27%). With regard to macroscopic type, type III was the most common macroscopic type (61.9%). With regard to histologic classification, pro (39.68%) and signet-ring cell carcinoma (48.41%) were remarkable. With regard to tumor staging, almost half patients (47.62%) were classified as stage IV.

**Table 1:** Clinicopathological characteristics of the YG.

Variable	Total (n = 126, %)
Age	20–40
Gender	
Male	68 (53.97)
Female	58 (46.03)
Family history of GC	9 (7.14)
Symptom classification	
Epigastric pain	94 (74.60)
Nausea/vomitin	40 (31.75)
Anemia/bleeding	26 (20.63)
Weight loss	32 (25.40)
Heartburn/acid reflux	33 (26.19)
Dyspepsia	74 (58.73)
Choking when eating	5 (3.97)
Tumor location	
Cardia	11 (8.73)
Non-cardia	115 (91.27)
Macroscopic types	
0 type	2 (1.59)
I type	9 (7.14)
II type	20 (15.87)
III type	78 (61.90)
IV type	17 (13.49)
Histological type	
pap	0 (0)
tub	15 (11.91)
muc	0 (0)
sig	61 (48.41)
por	50 (39.68)
Stage	
I	18 (14.29)
II	17 (13.49)
III	31 (24.60)
IV	60 (47.62)
Treatment method	
Curative resection	66 (52.38)
Uncurative resection	60 (47.62)
Recurrence	2 (1.59)

GC = gastric cancer, muc = mucinous adenocarcinoma, pap = papillary adenocarcinoma, pro = poorly differentiated adenocarcinoma, sig = signet ring cell carcinoma, tub = tubular adenocarcinoma, YG = young adult patients with gastric cancer (individuals <40 yr).

### 3.2. Treatment method

The treatment plan was made according to the tumor stage. Patients were divided into 2 groups: curative resection and non-curative resection (chemotherapy, palliative surgery or non-treatment). Curative resection includes endoscopic therapy and radical gastrectomy. Absolute indications for ESD (endoscopic submucosal dissection): a differentiated-type carcinomas in macroscopically intramucosal (cT1a) with a long diameter <2 cm with no ulceration or ulcer scar. Process of ESD with a dual knife for a lesion: Marking of the periphery of the lesion, 5 mm away from the lateral margin of the lesion. Injection of fluid into the submucosal layer. Mucosal incision started from the initial injection point and pushed toward the far side. Submucosal dissection with a dual knife. Dual knife treat potential bleeding points. Two patients met the endoscopic resection criteria, and were treated with ESD. These 2 patients had a curative resection. Principles of surgical treatment: resectable tumors, T1a-T3 tumors should be resected with a sufficient range of gastrectomy (at least 2-thirds of the stomach with a D2 lymph node dissection). Total (subtotal) or distal gastrectomy was performed according to the tumor size, location, status of resection margins and lymph node involvement. In surgical cases, curative resection was considered when microscopic examination revealed a negative resection margin. Sixty-four patients were treated with gastrectomy and get a curative resection. Sixty-six patients get a curative resection, and the overall resectable rate was 52.38%. Palliative surgery was considered when microscopic examination revealed a positive tumor margin or residual gross disease. Regimens of chemotherapy include 5-fluorouracil, oxaliplatin, tegafur, docetaxel, tegio and others. Sixty patients (47.62%) were treated with uncurative resection. Two patients (1.59%) who were treated with curative resection recurrented during follow-up.

### 3.3. Survival

**Figure 1.**
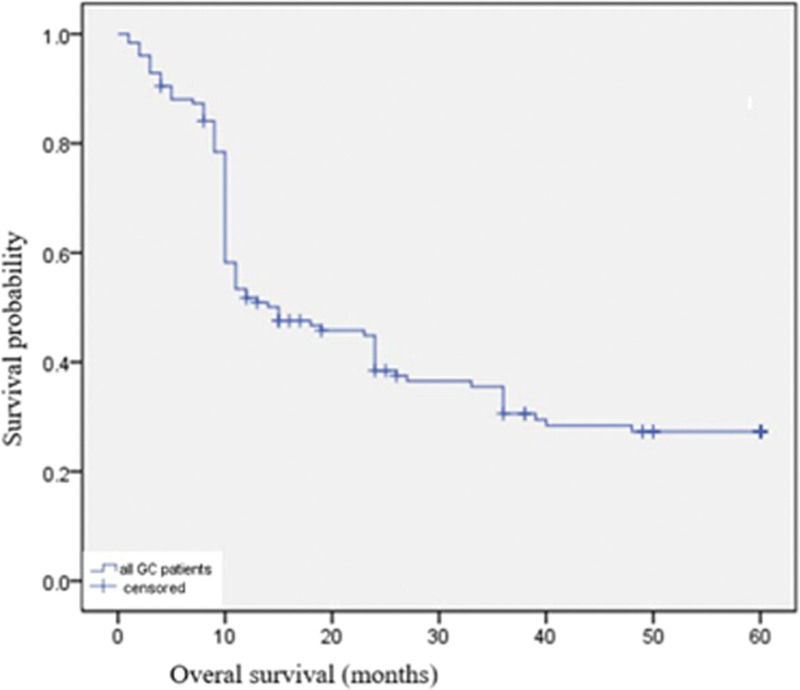
The 5-yr survival of all YG. The 5-yr survival of all YG was 31.7%. YG = young adult patients with gastric cancer (individuals <40 yr).

**Figure 2.**
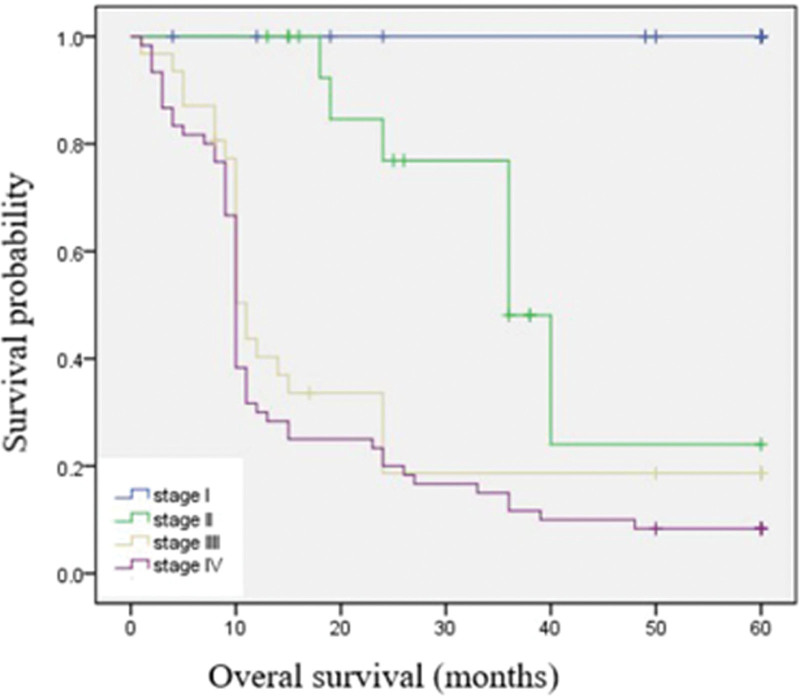
The 5-yr survival of YG differed from tumor staging. The 5-yr survival of YG was 100% in stage I group, 58.8% in stage II group, 22.6% in stage III group and 8.3% in stage IV group. YG = young adult patients with gastric cancer (individuals <40 yr).

**Figure 3.**
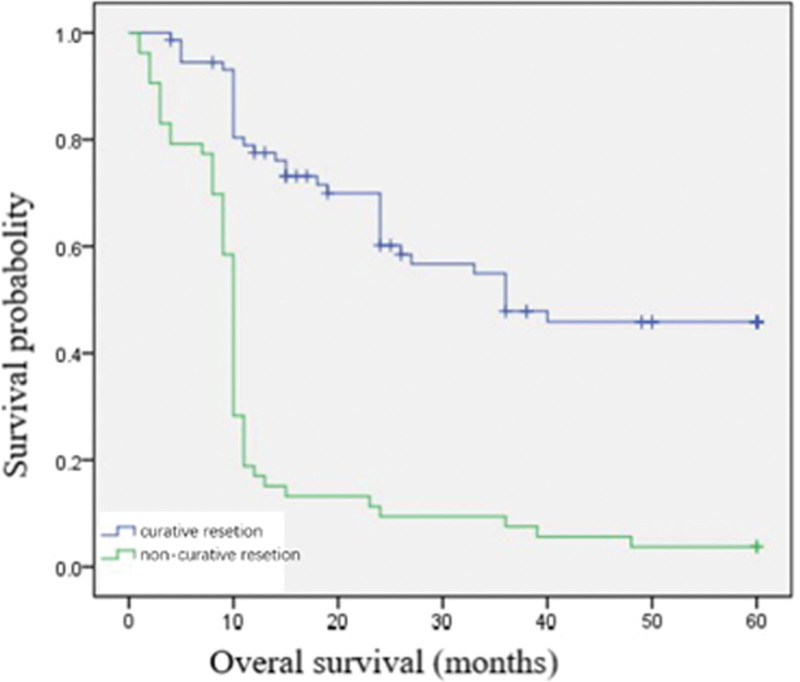
The 5-yr survival of YG differed from treatment methods. The 5-yr survival was 52.1% in curative resection group, while it was 3.8% in non-curative resection group. YG = young adult patients with gastric cancer (individuals <40 yr).

The overall median follow-up was 96.5 months (rang 1–192 months). Among our 126 patients, 86 patients (68.3%) died from disease-specific causes within 5 years since GC diagnosis and no patients died from other causes. The 5-year overall survival was 31.7% (Fig. 1). When the survival was determined according to the tumor stage, significant difference was found. The 5-year survival was 100% in stage I group, 58.8% in stage II group, 22.6% in stage III group, and 8.3% in stage IV group respectively (Fig. 2). When the survival was determined according to the treatment method, the 5-year survival was 52.1% in the curative resection group, while 3.8% in the non-curative resection group (Fig. 3).

### 3.4. Prognostic analysis

Univariate analysis Cox proportional hazards regression model was conducted based on gender, age, disease stage, macroscopic type, histology subtype, tumor localization, treatment method, family history and tumor recurrence. Male (*P* < .001), non-cardia tumor (*P* < .001), curative resection (*P* < .001), early tumor staging (*P* < .001) were significantly associated with better survival. Based on univariate analysis results, multivariate analysis using Cox proportional hazard model was performed. Tumor staging (*P* = .002) and treatment method (*P* = .034) were 2 independent prognostic factors of survival. Early tumor staging and curative resection may improve survival.

The details of Cox regression analysis of overall survival were shown in Table 2.

**Table 2:** Univariate and multivariate analysis of young GC patients.

	Univariate analysis		Multivariate analysis	
	HR (95%CI)	*P*	HR (95%CI)	*P*
Age	0.929 (0.887–0.972)	.002		
Sex				
Male	reference			
Female	1.581 (1.034–2.417)	.035		
Family history of GC				
No	reference			
Yes	0.784 (0.342–1.801)	.567		
Tumor location				
Cardia	reference			
Non-cardia	0.277 (0.145–0.527)	<.001		
Treatment method				
Curative resection	reference			
Non-curative resection	4.405 (2.810–6.905)	<.001	1.916 (1.051–3.491)	.034
Macroscopic types				
0 type	reference			
I type	-	.963		
II type	1.173 (0.477–2.885)	.729		
III type	0.346 (0.155–0.772)	.01		
IV type	0.649 (0.366–1.150)	.139		
Tumor staging				
I	reference			
II	172.965 (0.247–121123.344)	.123	1.730 (1.213–2.467)	.002
III	8.044 (1.525–42.426)	.014		
IV	3.649 (1.666–7.991)	.001		
Histological type				
Differenced*	reference			
Undifferenced†	1.124 (0.563–2.244)	.74		
Recurrence				
No	reference			
Yes	0.048 (0–12.618)	.285		

CI = confidence interval, GC = gastric cancer, HR = hazard ratio.

* Differentiated carcinomas include tubular adenocarcinomas and papillary adenocarcinomas.

† Undifferentiated carcinomas include poorly differentiated adenocarcinomas, signet ring cell carcinomas, and mucinous carcinomas.

## 4. Discussion

In this study, we summarized the clinicopathologic characteristics and analyzed the survival and prognosis of patients <40 years with GC. Most patients had clinical symptoms, such as epigastric pain (74.60 %) and dyspepsia (58.73 %) (have corrected). These tumors showed advanced stage at onset and an undifferentiated histologic tumor type feature. These features present here were comparable to other reports.^[6,7,18]^ Signet ring cell carcinoma and pro are classified as undifferentiated type category in the Japanese GC association,^[21]^ and diffuse-type cancer by Lauren.^[10]^ Undifferentiated adenocarcinoma characterized as rapid progression of disease and lymph node metastasis occurred even in the early stage.^[6,9,15]^ Advanced stage and aggressive biological behavior herald a poor prognosis. This might explain the low 5-year overall survival (31.7 %), in our patients (have corrected). When the 5-year survival rate was matched for tumor stage, it had a significant difference. The 5-year survival was 100% in stage I, while 8.3% in stage IV. Tumor in stage I could be curatively resected by using endoscopic therapy or radical gastrectomy, while patients in stage IV lost the chance of curative resection. Curative resection was the single most important factor determining outcome in patients with GC. The 5-year survival in the curative resection group (52.1%) was much higher than those in the non-curative resection group (3.8%). These findings suggest that although GC in young patients have an aggressive tumor biology, if it is diagnosed at an early stage and then proceed to be curatively resected, the prognosis is better. Our results show that nearly half of tumors (47.8%) in the YG were classified into stage IV at onset. It may be attributed to a lack of screening endoscopy in young population with mild symptom. Upper endoscopy for GC has been recommended for individuals 40 years and older.^[22]^ However, this screening criteria ignore populations younger than 40 years, thus, this subgroup might be overlooked, leading to a potential delay in diagnosis. For early diagnosis, populations younger than 40 years complies with clauses any one below should be listed as a high-risk group for GC, and it is recommended as a screening pair Elephant: People in areas with high incidence of GC; Helicobacter pylori infection; Malignant precancerous diseases of the stomach such as: chronic atrophic disease: Gastritis, gastric ulcer, gastric polyps, residual stomach after surgery, hypertrophic gastritis, anemia; First-degree relatives of GC patients; (5) Deposit Other risk factors for stomach cancer (high salt, pickled diet, smoking, severe alcohol consumption, etc).

Our prognostic analysis indicated that tumor stage (*P* = .002) and treatment method (*P* = .034) were 2 independent prognostic factors of survival. Early diagnosis and then proceed to a successful curative resection are vital for a better prognosis.

## 5. Limitation

First, our samples were fairly small, and this study was only in a single institution. Second, we only analyze the specific clinicopathologic characteristics in young patients with GC, not compare these characteristics with older patients.

## Acknowledgments

Thanks to colleagues in the pathology department of Liaocheng people Hospital and colleagues in the gastrointestinal surgery department of Liaocheng people Hospital.

## Author contributions

**Conceptualization:** Jina Lin, Peng Yan.

**Data curation:** Lifeng Liu.

**Formal analysis:** Jingrun Zhao.

**Investigation:** Jina Lin.

**Methodology:** Lifeng Liu.

**Writing – original draft:** Lifeng Liu.

**Writing – review & editing:** Jina Lin, Jingrun Zhao, Peng Yan.
